# Shaking culture enhances chondrogenic differentiation of mouse induced pluripotent stem cell constructs

**DOI:** 10.1038/s41598-020-72038-y

**Published:** 2020-09-14

**Authors:** Phoonsuk Limraksasin, Yukihiro Kosaka, Maolin Zhang, Naohiro Horie, Takeru Kondo, Hiroko Okawa, Masahiro Yamada, Hiroshi Egusa

**Affiliations:** 1 Division of Molecular and Regenerative Prosthodontics, Tohoku University Graduate School of Dentistry, 4-1 Seiryo-machi, Aoba-ku, Sendai, Miyagi 980-8575 Japan; 2 Weintraub Center for Reconstructive Biotechnology, UCLA School of Dentistry, Los Angeles, CA 90095-1668 USA; 3 Center for Advanced Stem Cell and Regenerative Research, Tohoku University Graduate School of Dentistry, Sendai, Miyagi 980-8575 Japan

**Keywords:** Stem-cell biotechnology, Stem-cell differentiation

## Abstract

Mechanical loading on articular cartilage induces various mechanical stresses and strains. In vitro hydrodynamic forces such as compression, shear and tension impact various cellular properties including chondrogenic differentiation, leading us to hypothesize that shaking culture might affect the chondrogenic induction of induced pluripotent stem cell (iPSC) constructs. Three-dimensional mouse iPSC constructs were fabricated in a day using U-bottom 96-well plates, and were subjected to preliminary chondrogenic induction for 3 days in static condition, followed by chondrogenic induction culture using a see-saw shaker for 17 days. After 21 days, chondrogenically induced iPSC (CI-iPSC) constructs contained chondrocyte-like cells with abundant ECM components. Shaking culture significantly promoted cell aggregation, and induced significantly higher expression of chondrogenic-related marker genes than static culture at day 21. Immunohistochemical analysis also revealed higher chondrogenic protein expression. Furthemore, in the shaking groups, CI-iPSCs showed upregulation of TGF-β and Wnt signaling-related genes, which are known to play an important role in regulating cartilage development. These results suggest that shaking culture activates TGF-β expression and Wnt signaling to promote chondrogenic differentiation in mouse iPSCs in vitro. Shaking culture, a simple and convenient approach, could provide a promising strategy for iPSC-based cartilage bioengineering for study of disease mechanisms and new therapies.

## Introduction

Induced pluripotent stem cells (iPSCs), which can be generated by reprogramming of somatic cells, have unlimited self-renewal capacity and pluripotency^1^. Because of these intrinsic properties, iPSCs are expected to be a new chondrocyte source for therapeutic application in cartilage injuries and disorders^2^. In addition, iPSCs possess high proliferation capacity and self-organizing potential, and thus can differentiate and form three-dimentional (3D) cartilaginous particles^3^. Because in vitro models with a 3D structure are more representative of the physiolosical condition, 3D cartilage tissue/organoids bioengineered using iPSCs would be well-suited for the study of unknown disease mechanisms and new treatments^4^. However, to date there are few published studies regarding therapeutic applications of iPSCs in cartilage diseases, possibly because robust and reproducible protocols to fabricate cartilaginous tissues from iPSCs have not yet been established^5^. Some methods have been reported to achieve chondrogenic induction of 3D cultures of iPSCs; however, these methods are time-consuming and require complicated procedures^3,6,7^. Therefore, chondrogenic differentiation of iPSCs awaits effective protocols to rapidly guide the differentiation of the iPSCs toward chondrogenic cells or tissue.

Mechanical loading on articular cartilage induces various mechanical stresses and strains including compression, shear and tension^8^. Alterations in joint loading stimulate cellular responses, which are either catabolic or anabolic depending on the impact type of the loading^9^. These mechanical stimuli considerably affect the differentiation of chondroprogenitor cells and chondrocytes^10^. Chondrogenic cells convert physical forces into biochemical signals through various mechanotransduction mechanisms associated with cellular components, such as tyrosine kinase receptors, ion channels, and various cytoskeletal filaments^11,12^. For instance, the MAP kinase/ERK signaling pathway has been recognized as a major mechanotransducer^13^. Hydrostatic pressure application inhibits Na^+^/K^+^ ion channels and activates Na^+^/H^+^ channels in chondrocytes through Na^+^ and H^+^ exchange activity^14^. Fluid flow shear stress enhances chondrocyte proliferation via activation of ion channels through Indian hedgehog and bone morphogenetic protein signaling pathways^15^. In human chondrocytes, α5β1 integrin serves as a mechanoreceptor that transmits mechanical forces by cyclic pressure from extracellular matrix (ECM) to cytoskeletal filaments^16^. Therefore, application of mechanical stimuli by bioreactors in chondrogenic differentiation has been extensively studied with regard to both basic and translational research aspects^17^.

Many studies have demonstrated that compression enhances chondrogenesis of mesenchymal stem cells (MSCs) and chondrocytes^18,19^, although the response is highly dependent on the frequency and duration of loading. Additionally, combination of shear with dynamic compression increases the chondrogenesis of these cells^18,19^. At the articular cartilage surface, shear stress generated by synovial fluid flow can be sensed by the cell-surface proteoglycan/glycoprotein layer for cartilage biosynthesis and remodeling^20,21^. These reports indicate that appropriate hydrodynamic stress is an important biophysical factor regulating the chondrogenic differentiation of chondroprogenitor cells; however, its effects on iPSCs during chondrogenic differentiation remain poorly understood.

Recently, various 3D cell culture methods have been developed to mimic the local environment of living tissues in vitro. Dynamic culture for these 3D cell culture methods convectively exposes cells to mechanical stimulation and transports nutrients^22^. Hydrodynamic forces impact various cellular properties at the single-cell, cell aggregate, and tissue level^23^. For instance, shaking culture methods provide noncontact hydrodynamic stimulation to cells^24^ and improve cellular activity^25^ and function^22,26^. We previously applied a gentle shaking culture method to mouse iPSC aggregates using a simple see-saw shaker during osteogenic induction, which generated osseous-like tissue in vitro^27^. However, to date, there has been no report of the use of shaking culture methods for chondrogenic induction of iPSCs.

Based on this background, we hypothesized that dynamic culture by shaking culture devices at specific frequencies would accelerate chondrogenic differentiation. To test this hypothesis, a shaking 3D culture method using a simple see-saw shaker was applied to the chondrogenic induction of mouse iPSCs. The objective of this study was to investigate effects of gentle shaking on chondrogenic induction of mouse iPSCs.

## Results

### Shaking culture influenced the size and aggregation profile of chondrogenically induced iPSC (CI-iPSC) constructs

**Figure 1:**
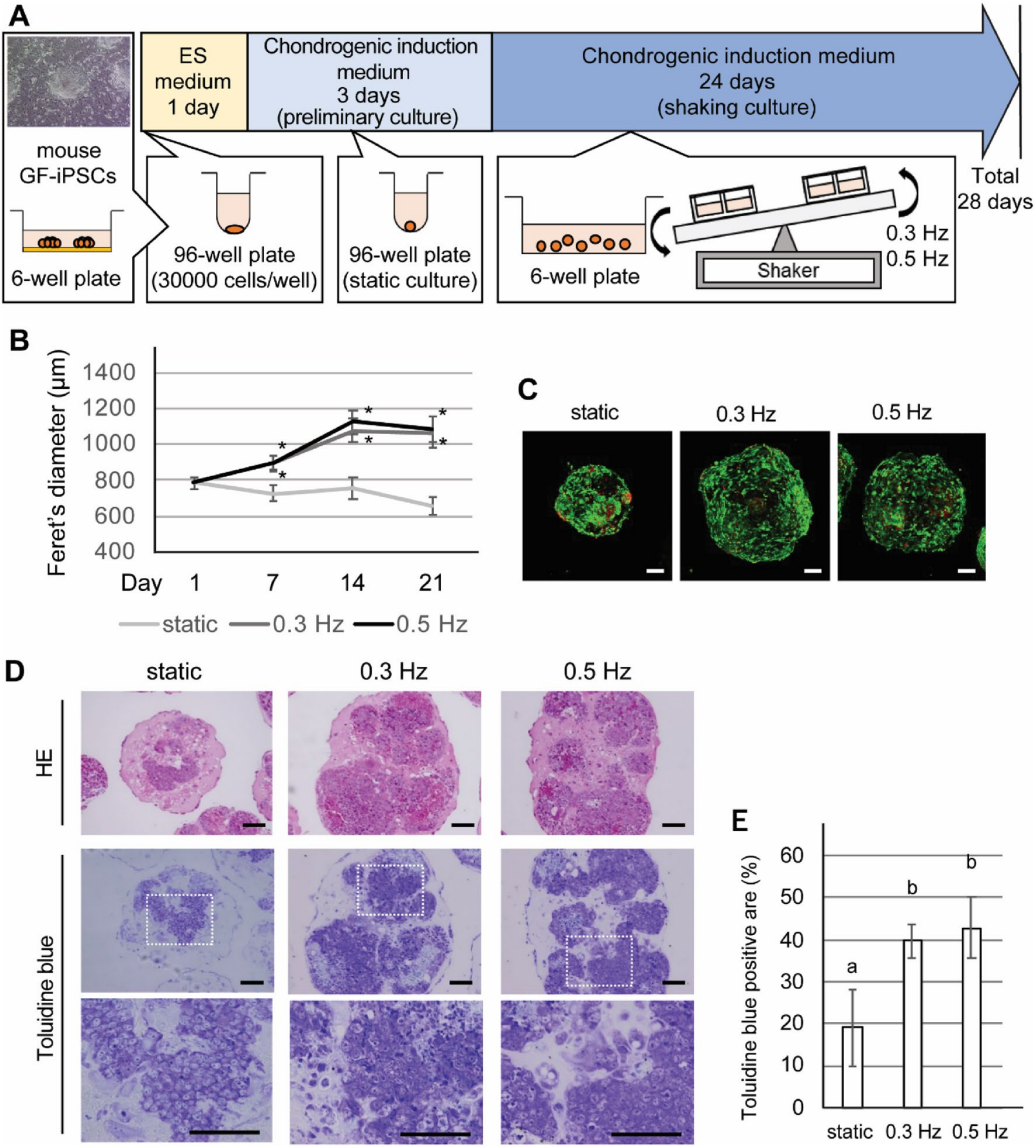
Effects of a shaking culture on chondrogenically induced iPSC (CI-iPSC) construct aggregation profiles. (**A**) Fabrication of CI-iPSC constructs. (**B**) The size of 5 randomly selected CI-iPSC constructs was measured using ImageJ software on culture days 1, 7, 14 and 21. Experiments were repeated three times with similar results. Representative data from three independent experiments are shown (mean values ± SD: n = 5). Asterisks indicate statistically significant differences with respect to the static group (*P* < 0.01, Dunnett’s correction for multiple comparisons). (**C**) Representative fluorescence images of live/dead (green/red) cells at a depth of approximately 200 µm from the surface of the 21-day CI-iPSC constructs under the static and shaking conditions (0.3 and 0.5 Hz). Scale bars; 100 μm. (**D**) Representative histological image of HE staining and toluidine blue staining obtained from 21-day cultures under the static and shaking conditions (0.3 and 0.5 Hz). Scale bars; 100 μm. (**E**) Quantitative analysis of toluidine blue-positive area. Experiments were performed in quintuplicate (5 different constructs) and repeated three times with similar results. Representative data from three independent experiments are shown (mean values ± SD: n = 5). Different letters indicate significant differences between groups (*P* < 0.01, ANOVA with Tukey’s multiple comparison test).

Mouse iPSCs were added to low-cell-attachment 96-well U bottom plates and maintained for 24 h (Fig. 1A). To induce chondrogenic differentiation, the medium was changed to chondrogenic induction medium. After 3 days, cell aggregates were transferred to low-cell-attachment plates and subjected to the suspension shaking culture in chondrogenic induction medium using a see-saw shaker^27^ at a frequency of 0 (static culture), 0.3 or 0.5 Hz (shaking culture). CI-iPSC constructs cultured in the shaking condition were significantly larger than those cultured in the static condition by day 21 (*P* < 0.01: Fig. 1B). The size of the initial aggregagtes observed at day 1 was 780 μm. After 7 days, the size of the CI-iPSC constructs increased, and it further increased to 1.1 mm by day 21 in the shaking culture groups at both 0.3 and 0.5 Hz. In contrast, the size of CI-iPSC constructs gradually decreased to 660 μm by day 21 in the static culture (Fig. 1B).

Viable cells in 21-day chondrogenically induced constructs were evaluated using the live/dead cell viability assay. Different sizes of CI-iPSC constructs were observed between the shaking and static culture groups. Although the evaluation of cellular viability was technically limited by the laser penetration depth to a depth of 200 µm from the surface of the constructs, the observed areas mostly contained live cells as indicated by green fluorescence; dead cells identified by red fluorescence were in the minority. This characteristic was present in both static and shaking culture groups (Fig. 1C).

We evaluated chondrogenic features in the 21-day CI-iPSC constructs using histological staining. Hematoxylin and eosin (HE) staining revealed a larger amount of cell clusters surrounded by ECM in the shaking culture compared to the static culture (Fig. 1D). Positive toluidine blue staining was observed in the cells and sparsely in the ECM in the shaking culture group, whereas such positivity was rarely observed in the static group. In addition, high-magnification images of the toluidine blue staining showed groups of purple, round cells located in lacunae in the shaking culture group but not in the static group. The toluidine blue-positive area was quantitatively analyzed to confirm that the shaking culture enhanced the chondrogenesis of CI-iPSCs (Fig. 1E). The toluidine blue-positive area was significantly increased when the cells were cultured in the shaking condition compared to the static condition (*P* < 0.01: Fig. 1E).

### Shaking culture promoted chondrogenic differentiation of iPSC constructs

**Figure 2:**
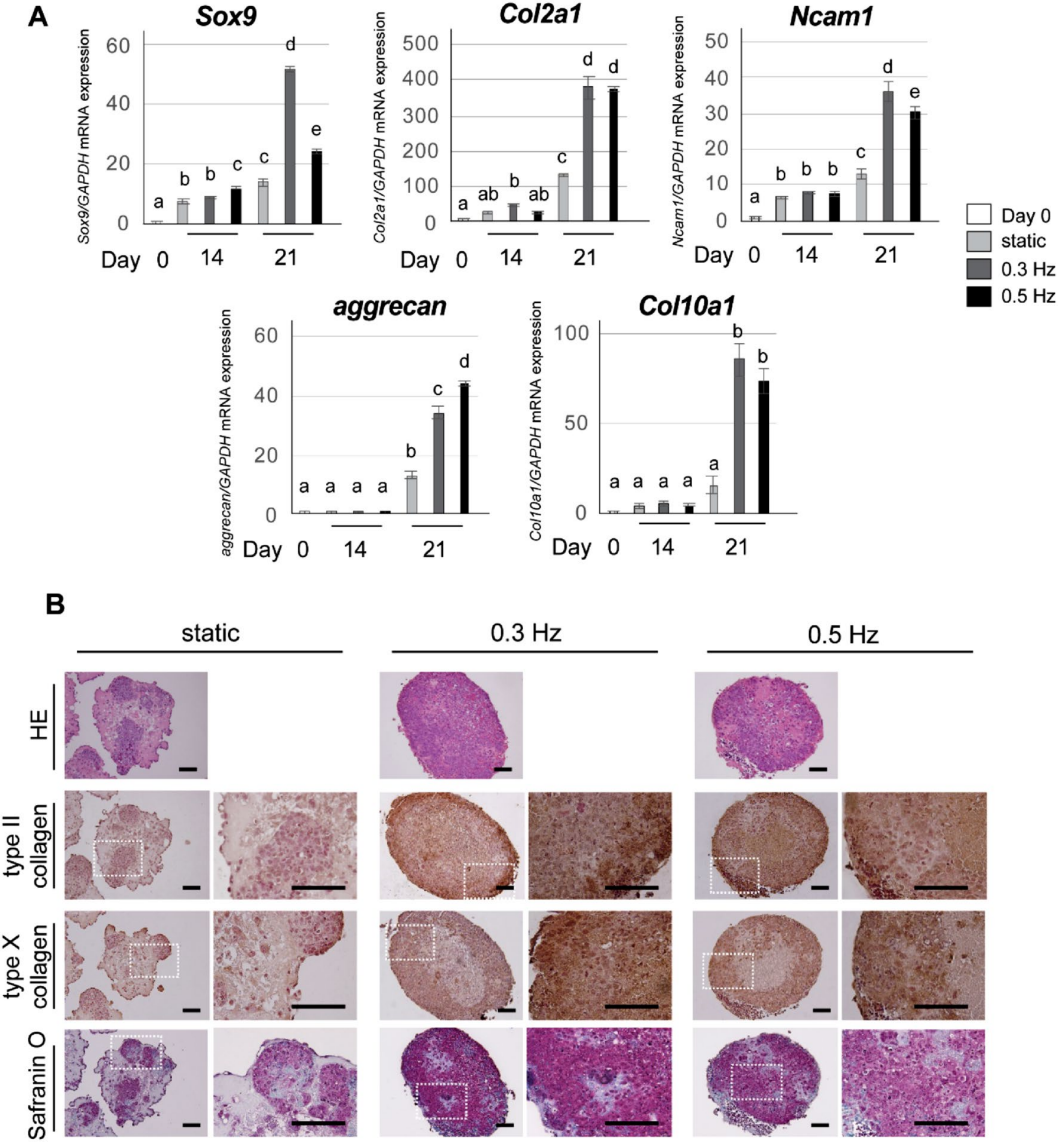
Effects of shaking culture on chondrogenic differentiation of CI-iPSC constructs. (**A**) Chondrogenic marker gene expression in static and shaking culture conditions. Shaking condition: 0.3 and 0.5 Hz. Gene expression of *Sox9*, *Collagen 2a1* (*Col2a1*), *Ncam1*, *aggrecan and Collagen 10a1* (*Col10a1*) was determined by real-time RT-PCR at days 0, 14 and 21. Experiments were performed in triplicate and repeated three times with similar results. Representative data from three independent experiments are shown (mean values ± SD: n = 3). Different letters indicate significant differences between groups (*P* < 0.01, ANOVA with Tukey’s multiple comparison test). (**B**) Representative histological images of CI-iPSC constructs in static and shaking culture conditions (0.3 and 0.5 Hz). CI-iPSC constructs at day 21 were stained with HE and Safranin O, and assessed by immunohistochemical detection of type II collagen and type X collagen. Scale bars; 100 μm.

We next studied the effects of shaking culture on chondrogenic marker gene expression. The shaking culture significantly promoted the expression of the transcription factor *Sox9* and chondrogenic-ECM related genes including *collagen 2a1* (*Col2a1*), *neural cell adhesion molecule-1* (*Ncam1*), *aggrecan* and *collagen 10a1* (*Col10a1*) by 21 days of chondrogenic induction (*P* < 0.01: Fig. 2A). In particular, shaking culture strongly enhanced the expression of these genes at day 21. There was no significant difference in the expression of *Col2a1* and *Col10a1* between the 0.3 and 0.5 Hz conditions at day 21. A shaking frequency of 0.3 Hz induced higher expression of the *Sox9* and *Ncam1* genes than 0.5 Hz. In contrast, the frequency of 0.5 Hz resulted in higher *aggrecan* gene expression compared to 0.3 Hz.

HE staining showed a group of round cells in lacunae with chondrocyte morphology in each condition; these cells were positively stained by safranin O (Fig. 2B). The chondrocyte morphology in the safranin O-stained sections was more evident in the shaking culture groups than in the static culture condition. In addition, these cells stained positively for type II collagen and type X collagen (Fig. 2B). In contrast, only slight expression of type II collagen and type X collagen protein was detected in the static culture group (Fig. 2B). There was no clear difference in protein accumulation between the 0.3 and 0.5 Hz conditions.

### Maturation of chondrogenic phenotype of iPSC constructs by long-term shaking culture

**Figure 3:**
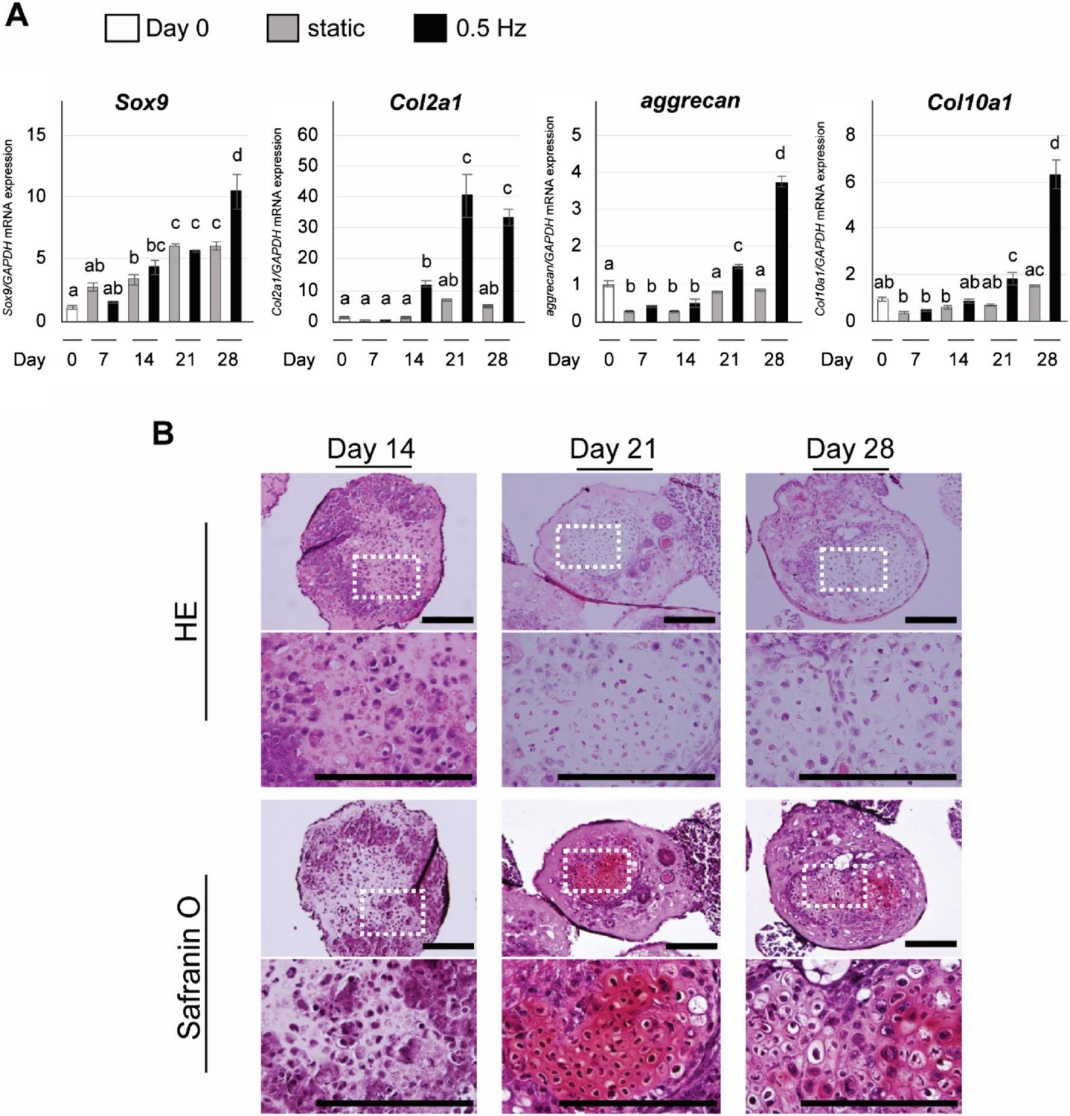
Evaluation of chondrogenesis in CI-iPSC constructs under long-term shaking culture. (**A**) Gene expression of *Sox9*, *Col2a1*, *aggrecan and Col10a1* was determined by quantitative real-time RT-PCR at days 0, 14, 21 and 28 under static and shaking conditions (0.5 Hz). Experiments were performed in triplicate and repeated three times with similar results. Representative data from three independent experiments are shown (mean values ± SD: n = 3). Different letters indicate significant differences between groups (*P* < 0.01, ANOVA with Tukey’s multiple comparison test). (**B**) Representative histological images of CI-iPSC constructs under shaking culture at 0.5 Hz. The second and fourth panels are magnifications of the dotted square regions. CI-iPSC constructs at days 14, 21 and 28 were stained with HE and Safranin O. Safranin O stained large rounded or oval cells resembling hypertrophic chondrocytes. Scale bars; 200 μm.

We next aimed to determine whether long-term shaking culture (0.5 Hz) further accelerates chondrogenesis to produce a mature chondrogenic phenotype. Real-time reverse transcription polymerase chain reaction (RT-PCR) showed that expression of chondrogenic marker genes in the static condition almost reached a plateau at day 21 (Fig. 3A). In contrast, further upregulation of these genes was confirmed at day 28 in the shaking condition. In particular, *Sox9* expression was significantly increased compared to that at day 0 by day 14 (*P* < 0.01: Fig. 3A), with a tenfold increase observed by day 28. *Col2a1* expression at day 14 was significantly increased by tenfold compared to that at day 0 (*P* < 0.01: Fig. 3A) and the increased reached 35 to 40 fold by day 28. In addition, *aggrecan* and *Col10a1* expression was significantly increased at 28 days of induction compared to other time points (*P* < 0.01: Fig. 3A).

HE staining and safranin O staining were performed to confirm cartilage tissue formation of the IC-iPSC constructs by long-term shaking culture. At day 14, HE staining showed round cells that were negative for safranin O and surrounded by ECM (Fig. 3B). After 21 days, many cells showed a hypertrophic chondrocyte-like appearance with a large cell body containing an ovoid nucleus (Fig. 3B). These cells were positively stained by safranin O dye. In addition, some parts of the CI-iPSC constructs at day 28 showed mature chondrocyte morphology with enlarged hypertrophic lacunae, resembling hypertrophic cartilage (Fig. 3B).

### Increased expression of TGF-β signaling- and Wnt signaling-related genes during chondrogenic differentiation of iPSC constructs under shaking culture condition

**Figure 4:**
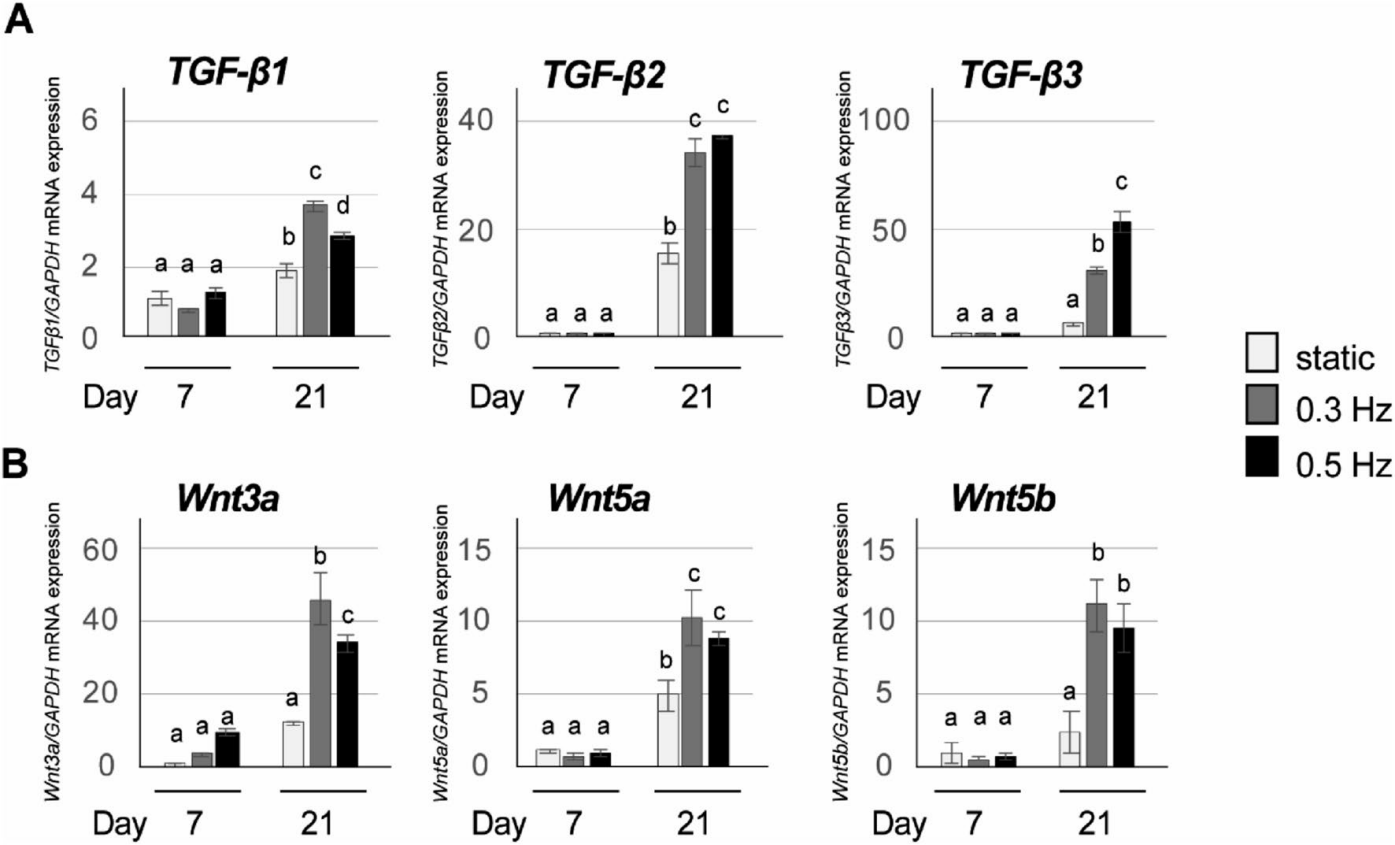
Involvement of TGF-β and Wnt signaling in shaking culture-enhanced chondrogenic differentiation of iPSC constructs. (**A**) Gene expression of *TGF-β1*, *TGF-β2* and *TGF-β3* and (**B**) *Wnt3a, Wnt5a,* and *Wnt5b* was determined by quantitative real-time RT-PCR at days 7 and 21. Shaking condition: 0.3 and 0.5 Hz. Experiments were performed in triplicate and repeated three times with similar results. Representative data from three independent experiments are shown (mean values ± SD: n = 3). Different letters indicate significant differences between groups (*P* < 0.01, ANOVA with Tukey’s multiple comparison test).

Previous reports showed that transforming growth factor (TGF)-β3 is a strong modulator of chodrocyte proliferation and maturation^28^. In addition, wingless-related integration site (Wnt) proteins including Wnt3a, Wnt5a and Wnt5b have been reported to be involved in chondrogenesis^29^. Therefore, we determined the effects of shaking culture on the expression of the TGF-β signaling-related genes *TGF-β1*, *TGF-β2* and *TGF-β3* as well as the Wnt signaling-related genes *Wnt3a*, *Wnt5a* and *Wnt5b* using real-time RT-PCR. Shaking culture significantly increased the gene expression of *TGF-β1*, *TGF-β2* and *TGF-β3* (*P* < 0.01: Fig. 4A) and *Wnt3a*, *Wnt5a* and *Wnt5b* (*P* < 0.01: Fig. 4B) at 21 days of chondrogenic induction.

### Effects of Wnt activation on chondrogenic differentiation of iPSC constructs under shaking culture

**Figure 5:**
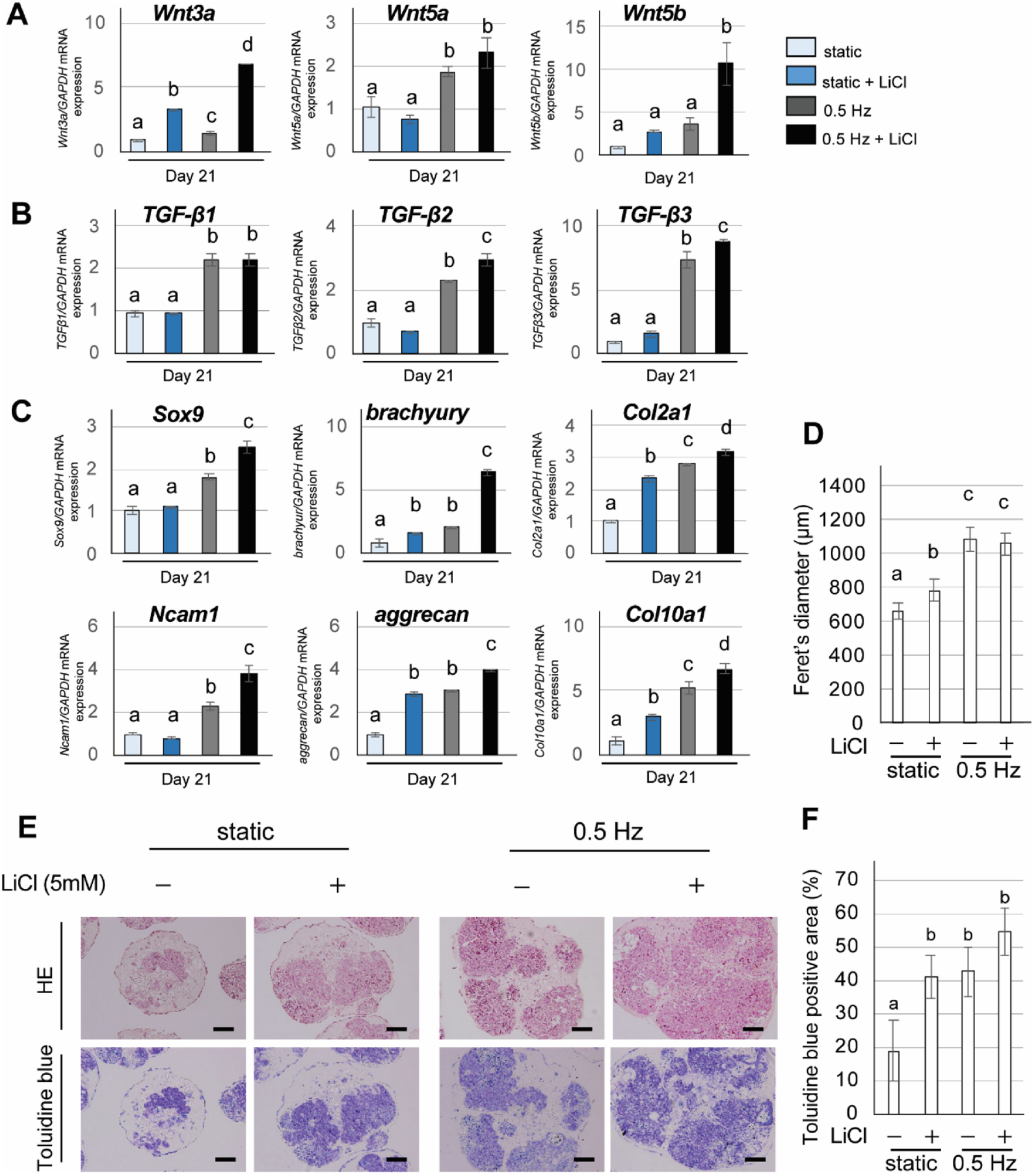
Effects of a Wnt signaling pathway activator (LiCl) on shaking culture-enhanced chondrogenic differentiation of iPSC constructs. iPSCs were subjected to chondrogenic differentiation under static or shaking (0.5 Hz) culture in the presence or absence of LiCl (5 mM). Gene expression of (**A**) *Wnt3a*, *Wnt5a* and *Wnt5b*, (**B**) *TGF-β1*, *TGF-β2* and *TGF-β3*, (**C**) *Sox9*, *brachyury*, *Col2a1*, *N-cam1*, *aggrecan* and *Col10a1* was determined by quantitative real-time RT-PCR at day 21. Experiments were performed in triplicate and repeated three times with similar results. Representative data from three independent experiments are shown (mean values ± SD: n = 3). Different letters indicate significant differences between groups (*P* < 0.01, ANOVA with Tukey’s multiple comparison test). (**D**) Size of CI-iPSC constructs at day 21. Experiments were performed in quintuplicate and repeated three times with similar results. Representative data from three independent experiments are shown (mean values ± SD: n = 5). Different letters indicate significant differences between groups (*P* < 0.01, ANOVA with Tukey’s multiple comparison test). (**E**) Representative histological image of HE staining and toluidine blue staining of CI-iPSC constructs at day 21. Scale bars; 100 μm. (**F**) Quantitative analysis of toluidine blue-positive area. Experiments were performed in quintuplicate (5 different constructs) and repeated three times with similar results. Representative data from three independent experiments are shown (mean values ± SD: n = 5). Different letters indicate significant differences between groups (*P* < 0.01, ANOVA with Tukey’s multiple comparison test).

To investigate the involvement of Wnt signaling in chondrogenic differentiation of iPSC constructs, lithium chloride (LiCl), a GSK-3 inhibitor, was applied to both static and shaking culture groups to activate Wnt signaling. At 21 days, the CI-iPSC constructs were collected for real-time RT-PCR and histological analyses. The gene expression of *Wnt3a*, *Wnt5a* and *Wnt5b* in the shaking groups was higher than that in the static group (Fig. 5A). Additionally, LiCl significantly increased the expression of these genes (*P* < 0.01), particularly in the shaking culture group. The gene expression of *TGF-β1*, *TGF-β2* and *TGF-β3* also showed a similar trend (Fig. 5B). The gene expression of *brachyury*, a mesodermal marker, and *Col2a1*, *aggrecan* and *Col10a1* was significantly increased by LiCl application under static culture (*P* < 0.01: Fig. 5C). Shaking culture significantly increased the expression of *Sox9*, *brachyury*, *Col2a1*, *Ncam1*, *aggrecan* and *Col10a1* (*P* < 0.01: Fig. 5C), and these genes were further up-regulated by LiCl treatment. In addition, LiCl treatment significantly augmented the size of 21-day CI-iPSC constructs under static culture (*P* < 0.01: Fig. 5D), whereas a significant difference was not observed between the LiCl-treated and non-treated groups under shaking culture (Fig. 5D). HE and toluidine blue staining showed a larger region of chondrogenic cells in LiCl-treated constructs under static and shaking culture (Fig. 5E,F). Toluidine blue staining showed round cells surrounded by ECM that were more evident in the LiCl-treated constructs than in the non-treated constructs (Fig. 5E). LiCl treatment in the static condition significantly increased the toluidine blue-positive area (*P* < 0.01: Fig. 5F) to the same level as in the shaking culture condition without LiCl treatment. Although LiCl treatment in the shaking condition apparently increased the positively stained area (Fig. 5F), the difference was not statistically significant.

### Effects of Wnt inhibition on chondrogenic differentiation of iPSC constructs under shaking culture

**Figure 6:**
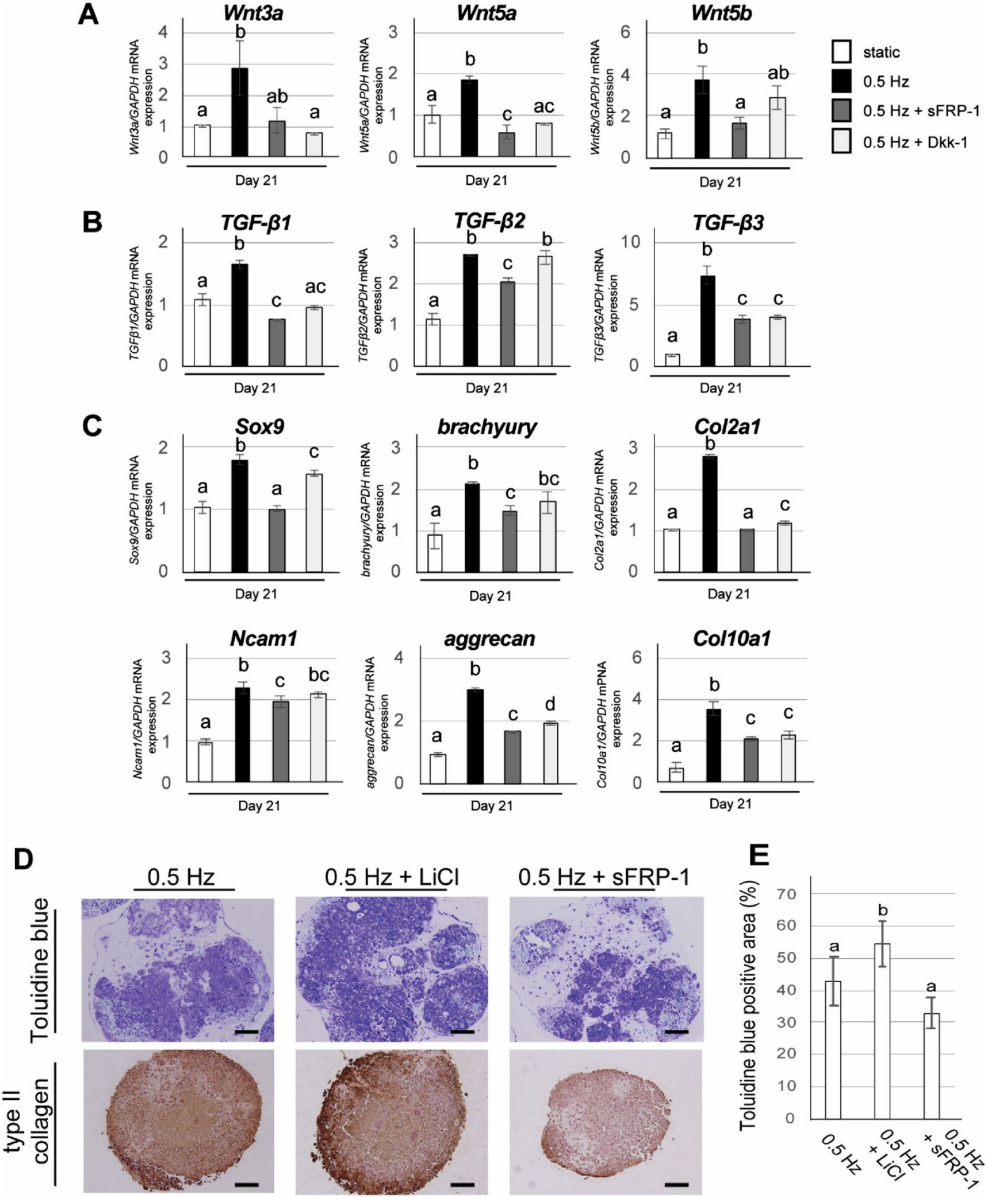
Effects of Wnt signaling pathway inhibitors (sFRP-1 and Dkk-1) and activator (LiCl) on shaking culture-enhanced chondrogenic differentiation of iPSC constructs. (**A–C**) iPSCs were subjected to chondrogenic differentiation under static or shaking (0.5 Hz) culture in the presence or absence of sFRP-1 (100 ng/ml) or Dkk-1 (100 ng/ml). Gene expression of (**A**) *Wnt3a*, *Wnt5a* and *Wnt5b*, (**B**) *TGF-β1*, *TGF-β2* and *TGF-β3*, (**C**) *Sox9*, *brachyury*, *Col2a1*, *N-cam1*, *aggrecan* and *Col10a1* was determined by quantitative real-time RT-PCR at day 21. Experiments were performed in triplicate and repeated three times with similar results. Representative data from three independent experiments are shown (mean values ± SD: n = 3). Different letters indicate significant differences between groups (*P* < 0.05 for *brachyury* and *Ncam1*, and *P* < 0.01 for other genes, ANOVA with Tukey’s multiple comparison test). (**D**, **E**) iPSCs were subjected to chondrogenic differentiation under shaking (0.5 Hz) culture in the presence or absence of LiCl (5 mM) and sFRP-1 (100 ng/ml). (**D**) Representative histological images of CI-iPSC constructs at day 21, which were subjected to toluidine blue staining and immunohistochemical detection of type II collagen. Scale bars; 100 μm. (**E**) Quantitative analysis of toluidine blue-positive area. Experiments were performed in quintuplicate (5 different constructs) and repeated three times with similar results. Representative data from three independent experiments are shown (mean values ± SD: n = 5). Different letters indicate significant differences between groups (*P* < 0.05, ANOVA with Tukey’s multiple comparison test).

To determine whether Wnt signaling was one of the underlying mechanisms for the shaking culture-mediated enhancement of chondrogenic differentiation, we next investigated the effects of the Wnt signaling inhibitors Dkk-1 and sFRP-1 during shaking culture. Wnt signaling initiates several cascades classified as either canonical or non-canonical, depending on whether β-catenin is involved^29^. Dkk-1 and sFRP-1 are inhibitors of only canonical Wnt pathways and both canonical and non-canonical Wnt pathways, respectively. Shaking culture significantly increased the expression of the *Wnt3a*, *Wnt5a* and *Wnt5b* genes (*P* < 0.01: Fig. 6A), and this increase was supressed by Dkk-1 and sFRP-1 treatment. CI-iPSC constructs also showed upregulation of *TGF-β1*, *TGF-β2* and *TGF-β3* genes in the shaking culture condition (Fig. 6B), and this upregulation, particularly that of the *TGF-β1*and *TGF-β3* genes, was suppressed by Dkk-1 and sFRP-1 treatment. Expression of the chondrogenic marker genes *Sox9*, *brachyury*, *Col2a1*, *Ncam1*, *aggrecan* and *Col10a1* was significantly increased by shaking culture (*P* < 0.01: Fig. 6C), and this increase was suppressed by Dkk-1 and sFRP-1 treatment, particularly for *Sox9*, *brachyury* and *Ncam1* with sFRP-1 treatment and for *Col2a1*, *aggrecan* and *Col10a1* with both sFRP-1 and Dkk-1 treatments (Fig. 6C).

Toluidine blue staining showed round cells positive for toluidine blue with chondrocyte morphology in the shaking culture group, both with and without LiCl (Fig. 6D). This chondrogenic morphology was rarely observed in the shaking condition with sFRP-1 treatment (Fig. 6D). In addition, immunohistochemical staining of type II collagen demonstrated round cells surrounded by type II collagen-positive ECM in the shaking culture with and without LiCl treatment (Fig. 6D). In contrast, type II collagen-negative ECM was observed in the sFRP-1-treated group (Fig. 6D). Additionally, LiCl treatment in the shaking culture significantly increased the toluidine blue-positive area (*P* < 0.05: Fig. 6E). In contrast, sFRP-1 treatment decreased the toluidine blue-positive area in the CI-iPSC constructs in the shaking culture, although the difference was not statistically significant (Fig. 6E). Fewer cells showed chondrocyte-like morphology in the CI-iPSC constructs with sFRP-1 treatment (Fig. 6D).

## Discussion

Chondrogenesis is an important process for the creation of chondrocytes during embryogenesis as well as during cartilage and bone repair in adult life^10^, and it is initiated by aggregation and condensation of loose mesenchyme. In this study, a simple see-saw shaking culture with a shaking frequency of 0.3 and 0.5 Hz was applied to CI-iPSC constructs. We selected shaking frequencies of 0.3 and 0.5 Hz in this study because the range of 0.1 to 1 Hz has been well established by previous mechanical loading studies for chondrogenic differentiation of MSCs^17^, but in our system, application of a frequency higher than 0.5 Hz was technically difficult due to medium overflow from the culture plates caused by the shaking. It should be noted, though, that most of the previous reports mentioned above applied compressive and shear forces to MSCs intermittently for short-term culture in a bioreactor^17^. We recently reported that osteogenic induction of mouse iPSCs using a micro-space culture method followed by simple see-saw shaking culture at 0.5 Hz generated bone-like tissue containing some cartilage-like tissue^30^. These observations prompted us to vary the shaking frequency to investigate the role of the shaking culture as well as underlying mechanisms on chondrogenic differentiation of iPSCs.

Shaking culture at 0.3 and 0.5 Hz significantly increased the size of CI-iPSC constructs compared to the static culture condition. Nii et al*.*^25^ reported that shaking culture using an orbital shaker with gelatin hydrogel microspheres increased the ATP levels and mitochondrial activity of cell aggregates of a mouse pre-osteoblast cell line, suggesting that shaking culture improved cellular viability. Other previous reports demonstrated that shaking culture improved the proliferation^22^ and function^26^ of MSCs because the dynamic motion of the medium effectively supplies oxygen and nutrients to the cell constructs, which may explain our findings.

In this study, shaking at 0.3 and 0.5 Hz enhanced the chondrogenesis of CI-iPSC constructs. Different mechanical strains could be induced by the shaking culture at the surface and the inner regions of the iPSC construct. Hydrodynamic shear stress and collision stress would be directly applied at the surface of the cell constructs, which would result in dynamic matrix and cell deformation from the surface to the inner core region with a gradient. Although strong evidence suggests the importance of fluid flow in the stimulation of chondrocyte biosynthesis^31^, it is largely unknown how fluid flow promotes chondrogenesis in the inner region of cartilage tissues. Previous reports showed that application of direct shear stress to cartilage explants without fluid flow significantly increased the synthesis of proteoglycans and proteins in both the core region and outer annular region of the explants comparably^32^. This suggests that matrix shear deformation is directly responsible for metabolic stimulation of the 3D cartilage tissue in a spatially independent manner^20^. Similarly, in our study, hydrodynamic force might have induced matrix shear deformation of the CI-iPSC constructs, thereby stimulating chondrogenesis in not only the outer region but also the inner region of the constructs. To understand the mechanistic complexity, it will be of interest to measure or simulate the mechanical stress distribution in the cell constructs during shaking culture.

We found that type II and X collagens were highly expressed in the outer region and that many hypertrophic cells were located in the inner region of the constructs, suggesting that region-specific microenvironments could differentially regulate chondrogenic maturation. Shaking culture might have increased the iPSC proliferation capacity and promoted ECM deposition in the cell constructs, resulting in increased size. A previous report using mouse embryonic stem cells (ESCs) indicated the possibility of cell interactions with neighboring cell constructs, and that a high density of cell constructs led to expression of mesodermal and endodermal genes^33^. Miyamoto et al*.*^34^ reported that differentiation commitment of mouse ESCs depends on the seeding cell number and size of cell constructs, which affect aspects of the microenvironment including hypoxia and cell-to-cell and construct-to-construct interactions. Hypoxic signaling is highly important for cartilage tissue engineering^29^. When iPSC constructs become large, particularly larger than 800 μm^35^, hypoxia occurs in the center of the constructs^36^. In our study, shaking culture increased the size of the cell constructs to approximately 1,000 μm at 21 days, and hypoxia should have thus occurred at the inner region of the constructs. It should be noted that most cells in the constructs were alive to a depth of at least 200 µm from the construct surface (200 µm was the maximum depth evaluated) during chondrogenesis, possibly because shaking culture facilitated oxygen perfusion into the cell aggregates. Sustained hypoxia enhances the induction of chondrogenesis with matrix synthesis^37^. We therefore hypothesize that appropriate hypoxia at the core of the large constructs together with dynamic mechanical stimulation by the fluid flow might have additively and/or synergistically improved the chondrogenesis of the iPSC constructs. Our method to induce chondrogenesis of iPSC constructs might thus represent an appropriate condition for cartilage engineering in light of the cell number and size of the constructs. Further studies are needed to clarify the effects of region-specific mechanical strain and hypoxia in the cell constructs generated by the shaking culture.

During the early developmental steps of chondrogenesis, the condensing mesenchyme expresses many ECM and adhesion molecules, including Ncam1^38^ and type II collagen^39^. In the present study, the CI-iPSCs in shaking culture at 0.3 and 0.5 Hz showed robust cell aggregates and higher expression of *Ncam1* and *col2a1* at day 21 compared to the static condition, implying enhanced condensation of CI-iPSC aggregates by shaking culture. Shaking culture significantly increased the expression of chondrogenic marker genes, including *Sox9*, *col2a1*, *aggrecan* and *col10a1* up to 28 days, which is representative of mesenchymal condensation-based chondrogenesis. Indeed, many cells exhibited a hypertrophic morphology and were positively stained by Safranin O after 21 days. The shaking culture also stimulated production of type II and type X collagens in CI-iPSC constructs. In agreement with our results, hydrodynamic stress has been shown to exert a significant effect on chondrocyte gene expression, tissue ECM metabolism and tissue mechanical properties^18^. These results suggest that the enhanced condensation of CI-iPSC aggregates induced by shaking culture accelerates chondrogenic differentiation through induction of chondrogenesis-related ECM molecules.

The essential transcription factor *Sox9*, which is broadly expressed in early chondrogenesis^40^, was consistently expressed in CI-iPSCs. In chondrogenic differentiation, *col10a1* is preferentially expressed in the late stage in prehypertrophic chondrocytes once cells withdraw from the cell cycle. In the present study, we observed a trend of high and simultaneous expression of *Sox9* and *col10a1* in the shaking culture condition at day 21, which appears contradictory. This might be accounted for by heterogeneity of the cell population in the CI-iPSC constructs, where the chondrogenic status of each cell varies. In addition, the expression cascades of chondrogenic marker genes in the transition from mesenchymal cells to chondrocytes and that from pluripotent cells to chondrocytes might be different, which may further explain the discordant expression pattern of early and late chondrogenic marker genes in CI-iPSCs.

Around the articular cartilage, hydrodynamic stress is generated by joint loading via fluid flow as an indirect biophysical factor; this stress is critically involved in chondrogenic regulation for normal cartilage homeostasis^17^. Chondroprogenitors have been shown to transduce and respond to a wide array of mechanical stimuli both during development and throughout adulthood^10^. Therefore, in addition to oxygen and nutrient permeation, mechanical stimulation by hydrodynamic fluid entrainment, pressurization and shear stress from the culture medium could also affect cellular activity^23^ during the shaking culture of CI-iPSC aggregates, particularly in chondrogenesis. In animal studies, moderate joint loading provides an anabolic response in chondrocytes^9,41^; therefore, the response of CI-iPSCs to nonaggressive mechanical loading may be necessary for improved cell viability and chondrogenesis. In this regard, shaking culture with a frequency of 0.3 and 0.5 Hz seems to be appropriate for maintenance of cell viability in CI-iPSC aggregates during condensation and chondrogenic differentiation.

To date, several studies have demonstrated induction of chondrogenic differentiation of mouse iPSCs; however, the induction methods used in previous reports are slow and require a long period of time. For example, Hontani et al*.*^6^ reported a method to induce chondrogenic differentiation of mouse iPSCs using 3D culture with an ultra-purified alginate gel, in which iPSCs were first guided to differentiate into MSCs by repeated passaging. Then, the cells were seeded on the alginate scaffold and subjected to chondrogenic differentiation for 28 days. Another method used cell sorting after 15 days of preliminary chondrogenic induction of mouse iPSCs, followed by pellet formation of the sorted cells and chondrogenic induction for an additional 21 days^42^. These methods are time-consuming and complicated, in contrast to the simple see-saw shaking culture method in this study, which facilitates rapid fabrication of cartilage tissue from mouse iPSCs.

Several signaling pathways are involved in chondrogenesis, including TGF-β signaling^43^ and Wnt signaling^44,45^. TGF-β is a multifunctional cytokine found in bone, cartilage matrix, and platelets, and it induces chondrocyte proliferation and maturation^28,46^. In this study, shaking culture significantly enhanced the expression of the *TGF-β1*, *TGF-β2* and *TGF-β3* genes at day 21. Mechanical stretching, such as shear stress, can increase TGF-β release and activation in renal and vascular cells^47^. Fluid flow and shear stress induce TGF-β signaling in epithelial and endothelial cells^48–50^. It is possible that in the present study, the hydrodynamic stress generated by shaking culture stimulated TGF-β signaling in CI-iPSCs to enhance chondrogenic differentiation.

The effects of shaking frequency on overall chondrogenic phenotype seemed to be equivalent between 0.3 and 0.5 Hz, or slightly more evident at 0.5 Hz with respect to aggrecan and TGF-β3 production, which are particularly important for chondrogenic phenotype. In human bone marrow-derived MSCs, TGF-β3 is more essential for chondrogenic induction than TGF-β1^51^. In this study, significantly higher gene expression of TGF-β3 was observed in the 0.5 Hz condition. Therefore, we selected the frequency of 0.5 Hz for the later parts of this study. Canonical Wnt signaling (also known as the Wnt/β-catenin pathway) has emerged as an important regulator of chondrogenic differentiation^44,52^. Wnt3a, Wnt5a and Wnt5b stimulate differentiation of chondrocytes but inhibit chondrocyte hypertrophy, an essential process in the later steps of chondrogenesis^29^. Cross-talk between Wnt and TGF-β has been shown to play an important role in development, although its role in the chondrogenic differentiation of iPSCs has rarely been investigated. TGF-β1-mediated chondrogenesis of mesenchymal progenitor cells involves Wnt signaling^53^. TGF-β3 induces chondrogenic differentiation of pericytes by activation of Wnt/β-catenin signaling^54^. In the present study, shaking culture significantly up-regulated *Wnt3a*, *Wnt5a* and *Wnt5b* at day 21. These results suggest that TGF-β signaling and Wnt signaling might affect each other during chondrogenesis of iPSCs.

We found that gentle shaking culture with an activator of Wnt signaling, LiCl (a GSK-3 inhibitor), promoted chondrogenesis in CI-iPSC aggregate cultures, as demonstrated by increased expression of chondrogenic marker genes as well as increased cell aggregate size and accumulation of sulfated proteoglycans in the matrix. These results extend previous studies showing that addition of GSK-3 inhibitors to human MSC chondrogenic culture promoted cartilage-specific gene expression and GAG accumulation^55,56^. It should be noted that the shaking condition without LiCl treatment resulted in significantly higher gene expression of *Sox9*, *Col2a1*, *Ncam1*, and *Col10a1* than the static condition with LiCl treatment. This implies that shaking loading promotes chondrogenic induction of mouse iPSC constructs through not only canonical Wnt signaling but also non-canonical Wnt signaling or other pathways including TGF-β signaling. Furthermore, these results suggest that combined use of the shaking culture and LiCl facilitates mature chondrogenic differentiation of iPSCs in 3D chondrogenic constructs without requiring the use of scaffolds. This technology could be advantageous for bioengineering of cartilage tissue in vitro given its simplicity, cost-effectiveness, and shortening of the culture period.

Inhibitors of Wnt signaling, sFRP-1 and Dkk-1, suppressed the shaking culture-induced expression of chondrogenic marker and TGF-β signaling genes and the deposition of type II collagen in the matrix. Previously, dynamic fluid flow was shown to induce mechanobiological modulation of calcium oscillation in osteocytes through canonical Wnt^57^. Shear stress has also been shown to activate canonical^58^ and non-canonical^59^ Wnt signaling in vascular epithelial cells, and to mediate the synthesis of the actin cytoskeleton and alter differentiation via canonical Wnt signaling in MSCs^60^. Our results suggest for the first time that shaking culture induces chondrogenic differentiation of mouse iPSCs through activation of Wnt and TGF-β signaling.

A limitation of the mechanistic elucidation in this study was that the gene expression analysis was based on total RNA samples collected from the whole iPSC construct, even though the 3D iPSC construct appeared to undergo region-specific chondrogenic differentiation. As mentioned above, two possible induction mechanisms might occur under the shaking culture condition: hydrodynamic force-derived matrix shear deformation predominantly in the outer region, and hypoxia in the inner region. Accordingly, chondrogenic differentiation in the iPSC constructs appears to be distinguished between a maturation stage in the outer region and a hypertrophy stage in the inner region. Nonetheless, it should be noted that TGF-β and canonical Wnt signaling is involved in all steps of chondrocyte differentiation, including maturation and hypertrophy^29,61–63^. Therefore, we consider that activation of TGF-β expression and Wnt signaling by the shaking culture might have contributed to the overall chondrogenic induction of the whole iPSC construct, in both the outer and inner regions, and that the evaluation of TGF-β and Wnt in the total bulk construct reflects this phenomenon. However, the mechanism at the level of specific zones or individual cells remains unclear. It will be of interest to determine whether zone-specific differentiation initiates zone-to-zone interactions to induce cartilage organoid formation as a whole.

Mechanically generated signals appear to play a critical role in the chondrogenic differentiation and maturation of iPSCs under shaking culture. Further optimization of procedures and knowledge of developmental biology should enable the establishment of cell differentiation protocols that yield fully functional chondrocyte-like cell populations as organoids. In this proof-of-principle study, we found that shaking culture of mouse iPSC constructs facilitates a chondrogenic and biosynthetic response that is advantageous for developing iPSC-based bioengineering. We anticipate that organoids produced using this technique could be applied to disease modeling and drug discovery platforms to better understand the mechanisms of cartilage diseases, and this system is also expected to be useful for cartilage regeneration and repair in the future.

In conclusion, the present study demonstrates that gentle shaking of mouse iPSC constructs significantly enhanced chondrogenesis, and the underlying mechanism likely involves activation of Wnt and TGF-β signaling. Wnt signaling activation using LiCl further enhanced chondrogenic differentiation in CI-iPSC constructs. Although further studies on the mechanism by which the shaking procedure affects Wnt and TGF-β signaling should be explored, scaffold-free CI-iPSC constructs prepared by shaking culture add new insights into normal physiology and diseases in cartilage tissues, and may provide a more suitable tissue replacement strategy to achieve functional cartilage regeneration.

## Methods

### iPSCs

Mouse gingival fibroblast-derived iPSCs, which we previously generated^64^, were used in this study. As described previously^65^, iPSCs were maintained on inactivated SNLP76.7–4 feeder cells in ES medium (DMEM with 4.5 g/L glucose and without sodium pyruvate; Nacalai Tesque, 15% FBS (Gibco/Life Technologies, Grand Island, NY, USA), 2 mM l-glutamine (Wako Pure Chemical), 1 × 10^–4^ M nonessential amino acids (Life Technologies, Grand Island, NY, USA), 1 × 10^–4^ M 2-mercaptoethanol (Life Technologies), 50 U of penicillin, and 50 µg/ml streptomycin (Wako Pure Chemical).

### Fabrication of iPSC constructs and in vitro chondrogenic induction (Fig. 1A)

Mouse iPSCs were expanded in ES medium on inactivated SNL76.7–4 feeder cells in 6-well culture plates. Then, iPSCs were dissociated to single cells with 0.25% trypsin (Wako Pure Chemical). Single cell suspensions in 100 µl of ES medium were added to low-cell-attachment 96-well U bottom plates (Greiner bio-one) with a concentration of 30,000 cells/well to fabricate iPSC constructs.

After 24 h of culture in ES medium, the medium was changed to chondrogenic induction medium containing high-glucose DMEM (Nacalai Tesque) supplemented with 10 ng/ml TGFβ-3 (Oncogene Research Products, Cambridge, MA), 100 nM dexamethasone (Sigma, St. Louis, MO), 50 µg/ml ascorbic acid (Sigma), 100 µg/ml sodium pyruvate (Sigma), 40 µg/ml L-proline (Sigma), and ITS-plus (Collaborative Biomedical Products, Cambridge, MA; final concentration: 6.25 µg/ml bovine insulin, 6.25 µg/ml transferrin, 6.25 µg/ml selenous acid, 5.33 µg/ml linoleic acid, and 1.25 µg/ml bovine serum albumin) for preliminary chondrogenic induction.

After 3 days of preliminary induction in 96-well plates, cell aggregates were transferred to low-cell-attachment 6-well plates (Thermo Fisher Science) and subjected to chondrogenic induction by suspension shaking culture in chondrogenic induction medium using a see-saw shaker (Invitro shaker Wave-SI; Code. 0054334-000, Taitec, Saitama, Japan)^27^. The Wnt activation group was incubated in chondrogenic induction medium, additionally supplemented with 5 mM LiCl (Wako, Pure Chemical), and the Wnt inhibition groups were incubated in chondrogenic induction medium additionally supplemented with 100 ng/ml sFRP-1 (R&D systems, Minneapolis, MN, USA) or 100 ng/ml Dkk-1 (R&D systems) during shaking culture. The culture medium was changed every 2 days.

### RT-PCR analysis

Cell pellets from the single-iPSC suspension (day 0) and CI-iPSC constructs collected at days 7, 14, 21 and 28 were subjected to TRIzol extraction (Ambion/Life Technologies, Carlsbad, USA). Total RNA isolation and purification were performed using a spin column (RNeasy Mini Kit; Qiagen, GmBH, Germany). Next, DNase treatment and removal were performed using a DNA-free kit (Invitrogen/Thermo Fisher Scientific, Vilnius, Lithuania). Complementary DNA was synthesized from 1 µg of total RNA. PCR analysis was performed on a StepOnePlus real-time PCR system (Applied Biosystems, Thermo Fisher Scientific, Waltham, MA, USA) using Thunderbird SYBR qPCR Mix (Toyobo, Osaka, Japan). Finally, the data were quantitatively analyzed using the comparative cycle time (*ΔΔ*CT) method. GAPDH was used as an internal control. The primer sequences are shown in Supplementary Table 1. Experiments were performed in triplicate, and further repeated three times independently using different batches of iPSCs.

### Size measurement and live/dead cell viability assay

The size of CI-iPSC constructs was evaluated using Feret’s diameter. In brief, images of CI-iPSC constructs in both static and shaking culture groups were obtained at days 1, 7, 14 and 21 using a phase contrast microscope. Subsequently, randomly selected 2-dimensional images of five CI-iPSC constructs were used for size measurement. Feret's diameter was analyzed using ImageJ software (National Institutes of Health). Experiments were performed in quintuplicate and independently repeated three times using different batches of iPSCs.

Cell viability was analyzed using the Live/Dead viability/cytotoxicity kit (Molecular Probes/Thermo Fisher Scientific). Calcein-AM and propidium iodide (PI) were used to identify live and dead cells, respectively. CI-iPSC constructs were collected at day 21 and washed with phosphate-buffered saline (PBS) prior to incubation with Live/Dead viability/cytotoxicity reagents for 30 min. Subsequently, fluorescence signals of the stained CI-iPSC constructs were three-dimensionally scanned (z-stack interval: 15–20 µm) using a fluorescence microscope (LSM780, Zeiss) from the surface down to 200 µm of laser penetration depth, with excitation/emission wavelengths of 494/517 nm and 528/617 nm to detect live (green) and dead (red) cells, respectively. The negative control condition was incubation of CI-iPSC constructs in 70% methanol for 30 min.

### Histological analyses

CI-iPSC constructs were fixed in 10% formalin neutral buffer solution (Wako Pure Chemical) for 1 day. Next, the specimens were embedded in paraffin. Sections were prepared with 8-μm thickness using a microtome. The sections were subjected to histological and immunohistochemical staining. Standard HE staining, toluidine blue staining and Safranin O staining were performed for the paraffin-embedded sections as previously described^30^.

### Histomorphometric quantification

A slide with toluidine blue staining was prepared for each group (day 21), containing one section each from approximately 15–25 IC-iPSC constructs in a 100×-magnified image. Five IC-iPSC constructs from each image were randomly selected, and the proportion of toluidine blue-positive area for the five different constructs was quantified using ImageJ software^30^. The toluidine blue-positive area was calculated as the ratio of the stained area (threshold: 105) to the whole construct area. The experiment was independently repeated three times using different batches of iPSCs.

### Immunohistochemical analyses

Immunohistochemical analyses were performed as previously described^30^, with some modifications. Before immunostaining, antigen retrieval was performed via incubation of specimens in 0.1% pepsin (Nacalai Tesque) in 0.5 M acetic acid (Sigma-Aldrich) at 37 °C for 1 h in a humid chamber. Deparaffinized sections were rehydrated and incubated in 0.3% hydrogen peroxide (H_2_O_2_; Wako Pure Chemical) in PBS to block endogenous peroxidase activity. Then, non-specific binding sites were blocked using 2% BSA (Wako Pure Chemical), 0.1% Tween20 (Sigma-Aldrich), and 0.01% Triton-X (Wako Pure Chemical) prior to incubation in the primary antibody at 4 °C overnight. Then, the samples were incubated in mouse IgGκ light chain binding protein conjugated to horseradish peroxidase (m-IgGκ BP-HRP: Santa Cruz Biotechnology, CA, USA) as the secondary antibody for 60 min. Next, the samples were washed with PBS and incubated in DAB substrate working solution (Roche Applied Science, Mannheim, Germany) for 5–15 min. Subsequently, hematoxylin was used to counterstain nuclei for visualization. The primary antibodies used in this study were an anti-type II collagen monoclonal antibody (M2139: Santa Cruz Biotechnology) and anti-type X collagen monoclonal antibody (X53: Invitrogen/Thermo Fisher Scientific).

### Statistical analyses

A one-way analysis of variance (ANOVA) with Tukey or Dunnett post hoc test was used for a comparison of more than two groups. Statistical significance was defined as *P* < 0.05.

## Supplementary information

Supplementary information

## Data Availability

The datasets generated and/or analyzed during the current study are available from the corresponding author on reasonable request.

## References

[1] Takahashi K, Yamanaka S (2006). Induction of pluripotent stem cells from mouse embryonic and adult fibroblast cultures by defined factors. Cell.

[2] Tsumaki N, Okada M, Yamashita A (2015). iPS cell technologies and cartilage regeneration. Bone.

[3] Yamashita A (2015). Generation of scaffoldless hyaline cartilaginous tissue from human iPSCs. Stem Cell Rep..

[4] Shi Y, Inoue H, Wu JC, Yamanaka S (2017). Induced pluripotent stem cell technology: a decade of progress. Nat. Rev. Drug Discov..

[5] Castro-Vinuelas R (2018). Induced pluripotent stem cells for cartilage repair: current status and future perspectives. Eur. Cell Mater..

[6] Hontani K (2019). Chondrogenic differentiation of mouse induced pluripotent stem cells using the three-dimensional culture with ultra-purified alginate gel. J. Biomed. Mater. Res. A.

[7] Lach MS (2019). Chondrogenic differentiation of pluripotent stem cells under controllable serum-free conditions. Int. J. Mol. Sci..

[8] Guilak F, Mow VC (2000). The mechanical environment of the chondrocyte: a biphasic finite element model of cell–matrix interactions in articular cartilage. J. Biomech..

[9] Arokoski JP, Jurvelin JS, Vaatainen U, Helminen HJ (2000). Normal and pathological adaptations of articular cartilage to joint loading. Scand. J. Med. Sci. Sports.

[10] Zuscik MJ, Hilton MJ, Zhang X, Chen D, O'Keefe RJ (2008). Regulation of chondrogenesis and chondrocyte differentiation by stress. J. Clin. Investig..

[11] Wang N, Tytell JD, Ingber DE (2009). Mechanotransduction at a distance: mechanically coupling the extracellular matrix with the nucleus. Nat. Rev. Mol. Cell Biol..

[12] Kwon H, Paschos NK, Hu JC, Athanasiou K (2016). Articular cartilage tissue engineering: the role of signaling molecules. Cell. Mol. Life Sci..

[13] Ryan JA, Eisner EA, DuRaine G, You Z, Reddi AH (2009). Mechanical compression of articular cartilage induces chondrocyte proliferation and inhibits proteoglycan synthesis by activation of the ERK pathway: implications for tissue engineering and regenerative medicine. J. Tissue Eng. Regen. Med..

[14] Browning JA, Walker RE, Hall AC, Wilkins RJ (1999). Modulation of Na^+^ × H^+^ exchange by hydrostatic pressure in isolated bovine articular chondrocytes. Acta Physiol. Scand..

[15] Wu Q, Zhang Y, Chen Q (2001). Indian hedgehog is an essential component of mechanotransduction complex to stimulate chondrocyte proliferation. J. Biol. Chem..

[16] Wright MO (1997). Hyperpolarisation of cultured human chondrocytes following cyclical pressure-induced strain: evidence of a role for alpha 5 beta 1 integrin as a chondrocyte mechanoreceptor. J. Orthop. Res..

[17] O'Conor CJ, Case N, Guilak F (2013). Mechanical regulation of chondrogenesis. Stem Cell Res. Ther..

[18] Anderson DE, Johnstone B (2017). Dynamic mechanical compression of chondrocytes for tissue engineering: a critical review. Front. Bioeng. Biotechnol..

[19] Fahy N, Alini M, Stoddart MJ (2018). Mechanical stimulation of mesenchymal stem cells: implications for cartilage tissue engineering. J. Orthop. Res..

[20] Grodzinsky AJ, Levenston ME, Jin M, Frank EH (2000). Cartilage tissue remodeling in response to mechanical forces. Annu. Rev. Biomed. Eng..

[21] Tarbell JM, Shi ZD (2013). Effect of the glycocalyx layer on transmission of interstitial flow shear stress to embedded cells. Biomech. Model. Mechanobiol..

[22] Yeatts AB, Fisher JP (2011). Bone tissue engineering bioreactors: dynamic culture and the influence of shear stress. Bone.

[23] Huber D, Oskooei A, Casadevall ISX, Andrew D, Kaigala GV (2018). Hydrodynamics in cell studies. Chem. Rev..

[24] Platas OB, Sandig V, Pörtner R, Zeng AP (2013). Evaluation of process parameters in shake flasks for mammalian cell culture. BMC Proc..

[25] Nii T, Makino K, Tabata T (2019). Influence of shaking culture on the biological functions of cell aggregates incorporating gelatin hydrogel microspheres. J. Biosci. Bioeng..

[26] Teixeira FG (2016). Modulation of the mesenchymal stem cell secretome using computer-controlled bioreactors: impact on neuronal cell proliferation, survival and differentiation. Sci. Rep..

[27] Okawa H (2016). Scaffold-free fabrication of osteoinductive cellular constructs using mouse gingiva-derived induced pluripotent stem cells. Stem Cells Int..

[28] Wang W, Rigueur D, Lyons KM (2014). TGFbeta signaling in cartilage development and maintenance. Birth Defects Res. C Embryo Today.

[29] Green JD (2015). Multifaceted signaling regulators of chondrogenesis: implications in cartilage regeneration and tissue engineering. Genes Dis..

[30] Limraksasin P (2020). In vitro fabrication of hybrid bone/cartilage complex using mouse induced pluripotent stem cells. Int. J. Mol. Sci..

[31] Zhao Z (2020). Mechanotransduction pathways in the regulation of cartilage chondrocyte homoeostasis. J. Cell. Mol. Med..

[32] Frank EH, Jin M, Loening AM, Levenston ME, Grodzinsky AJ (2000). A versatile shear and compression apparatus for mechanical stimulation of tissue culture explants. J. Biomech..

[33] Park J (2007). Microfabrication-based modulation of embryonic stem cell differentiation. Lab Chip.

[34] Miyamoto D, Ohno K, Hara T, Koga H, Nakazawa K (2016). Effect of separation distance on the growth and differentiation of mouse embryoid bodies in micropatterned cultures. J. Biosci. Bioeng..

[35] Van Winkle AP, Gates ID, Kallos MS (2012). Mass transfer limitations in embryoid bodies during human embryonic stem cell differentiation. Cells Tissues Organs.

[36] Miyamoto D, Nakazawa K (2016). Differentiation of mouse iPS cells is dependent on embryoid body size in microwell chip culture. J. Biosci. Bioeng..

[37] Coyle CH, Izzo NJ, Chu CR (2009). Sustained hypoxia enhances chondrocyte matrix synthesis. J. Orthop. Res..

[38] Widelitz RB, Jiang TX, Murray BA, Chuong CM (1993). Adhesion molecules in skeletogenesis: II. Neural cell adhesion molecules mediate precartilaginous mesenchymal condensations and enhance chondrogenesis. J. Cell. Physiol..

[39] Sandell LJ, Morris N, Robbins JR, Goldring MB (1991). Alternatively spliced type II procollagen mRNAs define distinct populations of cells during vertebral development: differential expression of the amino-propeptide. J. Cell. Biol..

[40] Akiyama H, Chaboissier MC, Martin JF, Schedl A, de Crombrugghe B (2002). The transcription factor Sox9 has essential roles in successive steps of the chondrocyte differentiation pathway and is required for expression of Sox5 and Sox6. Genes Dev..

[41] Griffin TM, Guilak F (2005). The role of mechanical loading in the onset and progression of osteoarthritis. Exerc. Sport Sci. Rev..

[42] Diekman BO (2012). Cartilage tissue engineering using differentiated and purified induced pluripotent stem cells. Proc. Natl. Acad. Sci. USA.

[43] Thielen NGM, van der Kraan PM, van Caam APM (2019). TGFβ/BMP signaling pathway in cartilage homeostasis. Cells.

[44] Usami Y, Gunawardena AT, Iwamoto M, Enomoto-Iwamoto M (2016). Wnt signaling in cartilage development and diseases: lessons from animal studies. Lab. Investig..

[45] Matta C, Zakany R (2013). Calcium signalling in chondrogenesis: implications for cartilage repair. Front. Biosci. (Schol. Ed.).

[46] Roelen BA, Dijke P (2003). Controlling mesenchymal stem cell differentiation by TGFBeta family members. J. Orthop. Sci..

[47] Cortes P, Riser B, Narins RG (1996). Glomerular hypertension and progressive renal disease: the interplay of mesangial cell stretch, cytokine formation and extracellular matrix synthesis. Contrib. Nephrol..

[48] Egorova AD (2011). Tgfbeta/Alk5 signaling is required for shear stress induced klf2 expression in embryonic endothelial cells. Dev. Dyn..

[49] Grabias BM, Konstantopoulos K (2013). Notch4-dependent antagonism of canonical TGF-beta1 signaling defines unique temporal fluctuations of SMAD3 activity in sheared proximal tubular epithelial cells. Am. J. Physiol. Renal Physiol..

[50] Kunnen SJ (2017). Fluid shear stress-induced TGF-beta/ALK5 signaling in renal epithelial cells is modulated by MEK1/2. Cell. Mol. Life Sci..

[51] Barry F, Boynton RE, Liu B, Murphy JM (2001). Chondrogenic differentiation of mesenchymal stem cells from bone marrow: differentiation-dependent gene expression of matrix components. Exp. Cell. Res..

[52] Wang X, Cornelis FMF, Lories RJ, Monteagudo S (2019). Exostosin-1 enhances canonical Wnt signaling activity during chondrogenic differentiation. Osteoarthritis Cartilage.

[53] Tuli R (2003). Transforming growth factor-beta-mediated chondrogenesis of human mesenchymal progenitor cells involves N-cadherin and mitogen-activated protein kinase and Wnt signaling cross-talk. J. Biol. Chem..

[54] Kirton JP, Crofts NJ, George SJ, Brennan K, Canfield AE (2007). Wnt/beta-catenin signaling stimulates chondrogenic and inhibits adipogenic differentiation of pericytes: potential relevance to vascular disease?. Circ. Res..

[55] Eslaminejad MB, Karimi N, Shahhoseini M (2013). Chondrogenic differentiation of human bone marrow-derived mesenchymal stem cells treated by GSK-3 inhibitors. Histochem. Cell. Biol..

[56] Tanthaisong P (2017). Enhanced chondrogenic differentiation of human umbilical cord Wharton's jelly derived mesenchymal stem cells by GSK-3 inhibitors. PLoS ONE.

[57] Hu M, Tian GW, Gibbons DE, Jiao J, Qin YX (2015). Dynamic fluid flow induced mechanobiological modulation of in situ osteocyte calcium oscillations. Arch. Biochem. Biophys..

[58] Goddard LM (2017). Hemodynamic forces sculpt developing heart valves through a KLF2-WNT9B paracrine signaling axis. Dev. Cell.

[59] Franco CA (2016). Non-canonical Wnt signalling modulates the endothelial shear stress flow sensor in vascular remodelling. Elife.

[60] Kuo YC (2015). Oscillatory shear stress mediates directional reorganization of actin cytoskeleton and alters differentiation propensity of mesenchymal stem cells. Stem Cells.

[61] Blaney Davidson EN (2009). Increase in ALK1/ALK5 ratio as a cause for elevated MMP-13 expression in osteoarthritis in humans and mice. J. Immunol..

[62] Hellingman CA (2011). Smad signaling determines chondrogenic differentiation of bone-marrow-derived mesenchymal stem cells: inhibition of Smad1/5/8P prevents terminal differentiation and calcification. Tissue Eng. Part A.

[63] Zhu M (2009). Activation of beta-catenin signaling in articular chondrocytes leads to osteoarthritis-like phenotype in adult beta-catenin conditional activation mice. J. Bone Miner. Res..

[64] Egusa H (2010). Gingival fibroblasts as a promising source of induced pluripotent stem cells. PLoS ONE.

[65] Egusa H (2014). Comparative analysis of mouse-induced pluripotent stem cells and mesenchymal stem cells during osteogenic differentiation in vitro. Stem Cells Dev..

