# Supplementary material for: Shaking culture enhances chondrogenic differentiation of mouse induced pluripotent stem cell constructs
Source: Sci Rep. 2020 Sep 14;10:14996. doi: 10.1038/s41598-020-72038-y (PMC7490351; doi:10.1038/s41598-020-72038-y)
Supplement: Supplementary file 1 — Supplementary information [file 41598_2020_72038_MOESM1_ESM.docx]

**Supplementary Information**

Shaking culture enhances chondrogenic differentiation of mouse induced pluripotent stem cell constructs

*Phoonsuk Limraksasin, Yukihiro Kosaka, Maolin Zhang, Naohiro Horie, Takeru Kondo, Hiroko Okawa, Masahiro Yamada, Hiroshi Egusa*

**Supplementary Table 1:** Primers used for SYBR Green real-time RT-PCR.

| Description  (gene name) | Primers (Fw, forward; Rv, reverse) | Product size (bp) | Accession number |
| --- | --- | --- | --- |
| *brachyury*  *(T)* | Fw: 5’-GCTTCAAGGAGCTAACTAACGAG-3’  Rv: 5’-CCAGCAAGAAAGAGTACATGGC-3’ | 117 | NM_009309.2 |
| *NCAM*  *(Ncam1)* | Fw: 5’-GGATGCCTCCATCCACCTC-3’  Rv: 5’-GGCCGTCTGATTCTCTACATAGG-3’ | 67 | NM_001081445.1 |
| *Sox9*  *(Sox9)* | Fw: 5'-CCTTCAACCTTCCTCACTACAGC-3'  Rv: 5'-GGTGGAGTAGAGCCCTGAGC-3' | 131 | NM_011448.4 |
| *Aggrecan*  *(aggerecan)* | Fw: 5'-CGCCACTTTCATGACCGAGA-3'  Rv: 5'-TCATTCAGACCGATCCACTGGTAG-3' | 146 | NM_007424.2 |
| *Collagen 2a1*  *(Col2a1)* | Fw: 5'-CCTCCGTCTACTGTCCACTGA-3'  Rv: 5'-ATTGGAGCCCTGGATGAGCA-3' | 121 | NM_001113515.2 |
| *Collagen 10a1*  *(Col10a1)* | Fw: 5'-GCCAAGCAGTCATGCCTGAT-3'  Rv: 5'-GACACGGGCATACCTGTTACC-3' | 106 | NM_009925.4 |
| *TGF-β1*  *(Tgfb1)* | Fw: 5'-TGCTTCAGCTCCACAGAGAA-3'  Rv: 5'-TGGTTGTAGAGGGCAAGGAC-3' | 182 | NM_011577.2 |
| *TGF-β2*  *(Tgfb2)* | Fw: 5'-TCGACATGGATCAGTTTATGCG-3'  Rv: 5'-CCCTGGTACTGTTGTAGATGGA-3' | 147 | NM_009367.4 |
| *TGF-β3*  *(Tgfb3)* | Fw: 5'-ATGACCCACGTCCCCTATCAG-3'  Rv: 5'-GCCAGTCCCTGGATCATGT-3' | 161 | NM_009368.3 |
| *Wnt3a*  *(Wnt3a)* | Fw: 5'-CTGGCAGCTGTGAAGTGAAG-3'  Rv: 5'-TGGGTGAGGCCTCGTAGTAG-3' | 210 | NM_009522.2 |
| *Wnt5a*  *(Wnt5a)* | Fw: 5'-CAAATAGGCAGCCGAGAGAC-3'  Rv: 5'-CTCTAGCGTCCACGAACTCC-3' | 217 | NM_009524.3 |
| *Wnt5b*  *(Wnt5b)* | Fw: 5'- GACTGACGCCAACTCCTG-3'  Rv: 5'- TGCTCCTGATACAACTGACAC-3' | 144 | NM_009525.3 |
| *GAPDH*  *(Gapdh)* | Fw: 5'- TGCACCACCAACTGCTTAG-3'  Rv: 5‘- GGATGCAGGGATGATGTTC-3' | 177 | NM_001289726.1 |
